# High-throughput sequencing of pooled samples to determine community-level microbiome diversity

**DOI:** 10.1016/j.annepidem.2019.09.002

**Published:** 2019-11

**Authors:** Kathryn J. Ray, Sun Y. Cotter, Ahmed M. Arzika, Jessica Kim, Nameywa Boubacar, Zhaoxia Zhou, Lina Zhong, Travis C. Porco, Jeremy D. Keenan, Thomas M. Lietman, Thuy Doan

**Affiliations:** a Francis I. Proctor Foundation, San Francisco; b UCSF Epidemiology and Biostatistics, University of California, San Francisco; c The Carter Center Niger, Republique du Niger, Niger; d UCSF Department of Ophthalmology, University of California, San Francisco

**Keywords:** Pooling, Cluster randomized trials, Gamma diversity, Microbiome diversity, Community-level diversity

## Abstract

**Purpose:**

Community-level interventions in cluster randomized controlled trials may alter the gut microbiome of individuals. The current method of estimating community diversities uses microbiome data obtained from multiple individual's specimens. Here we propose randomly pooling a number of microbiome samples from the same community into one sample before sequencing to estimate community-level microbiome diversity.

**Methods:**

We design and analyze an experiment to compare community microbiome diversity (gamma-diversity) estimates derived from 16S rRNA gene sequencing of 1) individually sequenced specimens vs. 2) pooled specimens collected from a community. Pool sizes of 10, 20, and 40 are considered. We then compare the gamma-estimates using Pearson's correlation as well as using Bland and Altman agreement analysis for three established diversity indices including richness, Simpson's and Shannon's.

**Results:**

The gamma-diversity estimates are highly correlated, with most being statistically significant. All correlations between all three diversity estimates are significant in the 10-pooled data. Pools comprising 40 specimens are closest to the line of agreement, but all pooled samples and individual samples fall within the 95% limits of agreement.

**Conclusions:**

Pooling microbiome samples before DNA amplification and metagenomics sequencing to estimate community-level diversity is a viable measure to consider in population-level association research studies.

## Introduction

Antibiotics given to an individual have been shown in longitudinal studies and a randomized controlled trial to decrease alpha-diversity in the gut microbiota [1]. Likewise, community-based interventions, such as mass distributions of antibiotics, may impact the gamma diversity of a community. Cluster randomized control trials (CRTs) are often more concerned with the collective effect of the intervention on the entire cluster rather than the effect at the individual level. Thus gamma diversity of a community is a plausible metric in the CRT setting. Gamma diversity is typically estimated using established ecological methods requiring taxa identification at the level of the individual host (e.g., animal or person). Individual processing combines results from different amplifications which could introduce bias, and individual processing is resource-intensive for microbiome studies [2,3]. One innovative way to estimate an intervention's impact on diversity in a community may be to pool samples taken from many individuals before sequencing rather than sequence each individual sample first.

Pooling of samples has long been used for screening for infection, although individuals from any positive-pool specimen would then need to be processed to identify the infected individual. Pooling has been used to assess community-level prevalence without the need to retest positive pools. The prevalence in the community most likely to have resulted in the results from the pools can easily be estimated [[4], [5], [6], [7]]. Human genetic researchers have pooled samples to identify changes in gene expression profiles, identify single-nucleotide polymorphisms, estimate allele frequencies, or identify genetic variants in genome-wide association studies [8,9]. Here, we estimate gamma diversity of a community using laboratory pooled specimens. Although pooling may seem intuitive in this context, variability may be introduced in several steps including collection of specimens, aliquot measurement, DNA amplification, and microbiota profiling [10,11]. We compare gamma diversity estimates using the established method of collecting and sequencing individual samples to pooling samples before sequencing, and assess whether pooling may be an efficient outcome for CRTs.

## Methods

### Collection of samples

This study used rectal swabs from a previously described RCT in Niger, of which children aged 1–60 months were assigned to receive oral azithromycin (approximately 20 mg per kilogram of body weight) or placebo 5 days before their rectal sample collection [1]. A total of 103 rectal swabs were obtained by trained field workers in the small community of Sarkin Yara Koira, Niger: 40 rectal swabs from children treated with azithromycin, 40 rectal swabs from children treated with placebo, 8 negative-control air swabs, and 15 positive-control duplicate rectal swabs. The health worker wore clean gloves each time he/she collected a rectal swab and placed the swab in a tube with Norgen Stool Nucleic Acid Preservative (Norgen Biotek Corp, Canada). The samples were stored as directed according to the manufacturer's recommendations for molecular studies.

### Sample preparation

Rectal swabs were randomized before DNA extraction and all laboratory personnel were masked to experimental characteristics associated with them including treatment assignment and/or pooling status. DNA isolation from swabs was done using Norgen Stool DNA Isolation Kit (Norgen Biotek Corp, Canada) as described previously [1]. Extracted DNA was quantified using QuBit and normalized. Samples for individuals were again randomized in order and relabeled with a new sample identification number before 16S rRNA gene deep sequencing at SeqMatic LLC (Fremont, CA).

### DNA sample construction

**Fig. 1:**
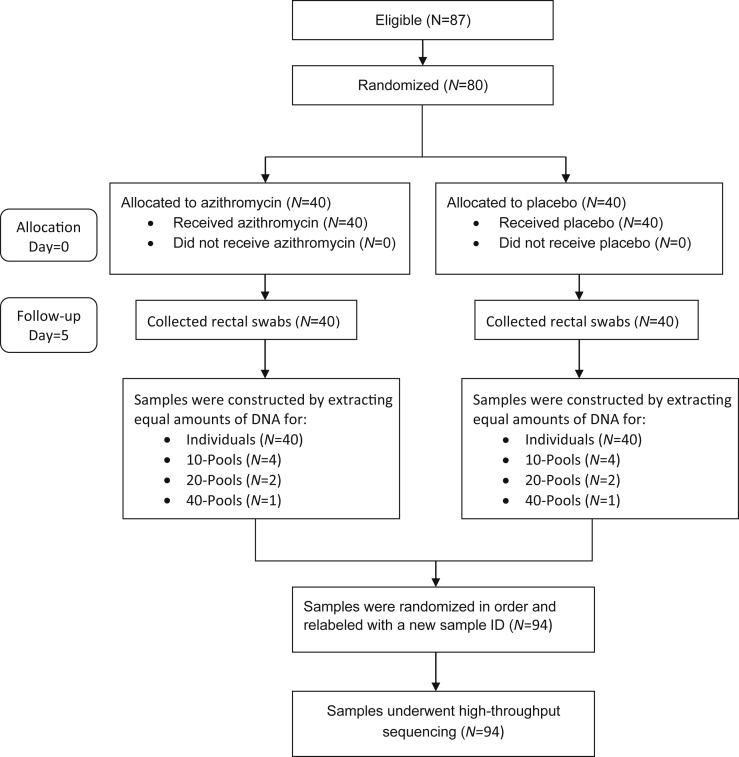
Flow diagram.

We considered four different methods for preparing samples from field specimens in our experiment: 1) eighty individual samples were constructed by extracting equal amounts of DNA, 2) eight 10-pools were constructed by extracting equal amounts of DNA using qubit from 10 rectal samples and then combined into a pooled sample, 3) four 20-pools were constructed by extracting equal amounts of DNA from 20 rectal samples and then combined into a pooled sample, and 4) two 40-pools were constructed by extracting equal amounts of DNA from 40 rectal samples and combined into a pooled sample. All samples were pooled together by treatment arm and component samples were randomly chosen for each of the three different pooling sizes. Here, because our objective is to validate an estimation method, we use the two treatment groups as separate populations because we know their diversities have been shown to be different previously [1]. Figure 1 summarizes the pooling assignments by treatment group. Once isolation of the DNA was completed and pooled samples were constructed, all samples were again randomized in order and relabeled with a new sample identification number before 16S rRNA gene deep sequencing at SeqMatic LLC (Fremont, CA).

### 16S rRNA gene deep sequencing

The gut microbiome was assessed by high-throughput sequencing of the V3-V4 hypervariable region of the 16S rRNA gene. Library preparation was performed per Illumina 16S metagenomic sequencing library preparation protocol and subjected to paired end Illumina sequencing on the MiSeq using paired 300 base-pair reads and MiSeq v3 reagents. Standard 16S metagenomic analysis was performed using Illumina's BaseSpace software which mapped the 16S reads against the GreenGenes database according to species or genus.

### Data analysis

We estimated diversity using a general class of diversity measures of order q equal to 0, 1, or 2, also known as Hill-numbers:

Dq=(∑i=1Spiq)1(1−q)

where S is the number of species, $p_{i}$ is the relative abundance of the species found in the sample [10,[12], [13], [14]]. In particular, we estimated diversity number equivalents using generalization of the classic species-neutral ecology diversity measures: species richness (D0), the exponential of Shannon entropy limq→1(D1) and the inverse Simpson concentration (D2) [12,13,15]. We considered estimation of gamma diversity using four different methods for preparing samples, individuals, 10-pools, 20-pools, and 40-pools. We used the mean gamma estimates when possible which included duplicate runs of individual specimens, repeated 40-pool construction, and combining 20- and 10-pool estimates by arm. We performed multiple rarefactions using the minimum sequencing depth from all 80 individual samples and *n* times this minimum sampling depth for the pooled samples, where *n* is the number of individual specimens contained in each pool. This ensured taxon relative abundances were equivalent, thus removing potential sample size biases which affect species richness and Shannon's diversity estimates [16]. All diversities were expressed in terms of the effective number, or the number of equal proportion taxa that would be equivalent to the same diversity as that observed in the unequal proportions of our sample [13].

We define our estimate of gamma diversity from pools here as the alpha diversity of the pooled sample. We compare this gamma estimate from pools to the traditional estimation of gamma diversity using individual samples [13]. Pearson's correlation coefficients and *P*-values were calculated for all possible combinations of pools except for 40-pools which only had two observations. Because gamma estimates using individual or pooled samples each produce some error in their measures, we check their agreement across ranges of diversity using the Bland and Altman limits of agreement using BlandAltmanLeh package in R [17]. All diversity calculations were conducted using the “vegetarian” package in R, version 3.3.3. Correlation of estimates of a community's γ -diversity based on pooled samples vs. individual samples for was calculated using Stata 13. Taxa for individual samples were rarefied using R.

## Results

### Richness (q = 0)

Table 1 displays the gamma diversity estimates by arm and sample preparation methods. The gamma diversity in the placebo arm was 350 genera among the samples sequenced from individual specimens (*n* = 80), 322 genera among the specimens pooled into groups of 10 (*n* = 4), 321 genera among the specimens grouped into pools of 20 (*n* = 2), and 342 genera among the samples grouped into pools of 40 (*n* = 1). For those children treated with azithromycin, average community richness was 332, 238, 265, and 305 among the individual, 10-pooled, 20-pooled, and 40-pooled samples, respectively (Table 1).

**Table 1:** Estimates of community diversity using four different methods for preparing samples from field specimens

Treatment arm	Method	Number of samples sequenced	Richness	Shannon's	Simpson's
			(Effective number)	(Effective number)	(Effective number)
Placebo	Individuals∗	40	350	36.0	18.3
	10-Pools	4	322	27.6	13.4
	20-Pools	2	321	27.3	12.4
	40-Pool∗	1	342	30.1	13.3
Antibiotics	Individuals∗	40	332	21.5	9.8
	10-Pools	4	238	14.4	7.3
	20-Pools	2	265	16.1	8.4
	40-Pool∗	1	305	16.3	7.7

Individual samples were constructed by extracting DNA from each of the 40 rectal samples. 10-Pools were constructed by combined DNA from 10 rectal samples into a pooled sample (*n* = 4/arm); 20-Pool were constructed by combined DNA from 20 rectal samples into a pooled sample (*n* = 2/arm); and 40-pools were constructed by combining DNA from 40 rectal samples into a pooled sample (*n* = 1/arm).

∗ Second aliquots were constructed for repeatability comparisons. Gamma estimates here are the average of the two different aliquots taken from the same specimens.

**Table 2:** Correlations between gamma estimates using pooled samples versus individual samples (established method)

Number of specimens	Richness (*q =* 0)				Shannon's (q = 1)				Simpson's (q = 2)				Tx†
	γ-diversity using pooled specimens	γ-diversity using individual specimens	ρ∗	*P*	γ-diversity using pooled specimens	γ-diversity using individual specimens	ρ∗	*P*	γ-diversity using pooled specimens	γ-diversity using individual specimens	ρ∗	*P*	
10	218	221	0.77	0.03	19.19	29.38	0.93	0.001	7.83	15.62	0.85	0.008	0
	199	241			33.23	35.30			17.22	18.29			0
	220	230			25.64	30.81			11.02	16.51			0
	211	227			31.48	31.65			17.32	18.10			0
	137	169			9.46	18.10			4.10	9.20			1
	172	222			18.99	22.22			10.70	10.17			1
	144	213			16.40	20.36			9.79	10.11			1
	157	192			12.19	15.71			4.79	6.40			1
20	245	282	0.98	0.02	26.84	34.48	0.94	0.06	12.50	17.98	0.81	0.19	0
	273	303			27.34	34.06			12.20	17.48			0
	224	268			20.06	22.11			11.27	10.77			1
	190	256			12.05	19.75			5.52	8.74			1
40	342	350	NA	NA	30.10	36.02	NA	NA	13.26	18.26	NA	NA	1
	305	332			16.29	21.54			7.71	9.80			0

All samples were rarefied to an equal number of sequence reads for comparisons.

∗ Pearson's correlation.

† 1 = treated with azithromycin; 0 = treated with antibiotics.

### Shannon's (q = 1)

The estimated Shannon's gamma diversity in the placebo arm was 36.0, 27.6, 27.3, and 30.1 in the individual, 10-pooled, 20-pooled, and 40-pooled samples, respectively. The estimated Shannon's γ -diversity in the azithromycin arm was 21.54, 14.35, 19.83, and 19.76 in the individual, 10-pooled, 20-pooled, and 40-pooled samples, respectively (Table 1).

### Simpson's (q = 2)

Using Simpson's gamma diversity, the sequenced taxa from the placebo arm produced 18.3, 13.4, 12.4, and 13.3 from the individual, 10-pooled, 20-pooled, and 40-pooled samples, respectively. Likewise, the estimated Simpson's gamma diversity in the azithromycin arm was estimated to be 9.8, 7.3, 8.4, and 7.7 from the individual, 10-pooled, 20-pooled, and 40-pooled samples, respectively (Table 1).

Table 2 shows the correlation coefficients between the gamma diversity estimates by arm and sample preparation methods. All correlations between all diversity estimates were significant in the 10-pool data (richness: ρ = 0.77, *P* = 0.03; Shannon's: ρ = 0.93, *P* = 0.001; Simpson's: ρ = 0.85, *P* = 0.008). In the 20-pool data, only richness gamma diversity estimates were correlated (richness: ρ = 0.98; *P* = 0.02). In the 40-pool data, because we only had two observations, Pearson's correlation coefficients were not reported. It should be noted that a Bonferroni multiple comparisons correction would require a *P*-value less than 0.0083 (*P* = 0.05/6 comparisons), which means only Shannon's and Simpson's 10-pools are significant after correcting for multiple comparisons. As a sensitivity analysis, nonrarefied reads were compared as well. Simpson's diversity measure is unbiased by sample size, so as expected, the estimates, correlation coefficients, and *P*-values remain essentially unchanged between the rarefied data and then nonrarefied data.

**Fig. 2:**
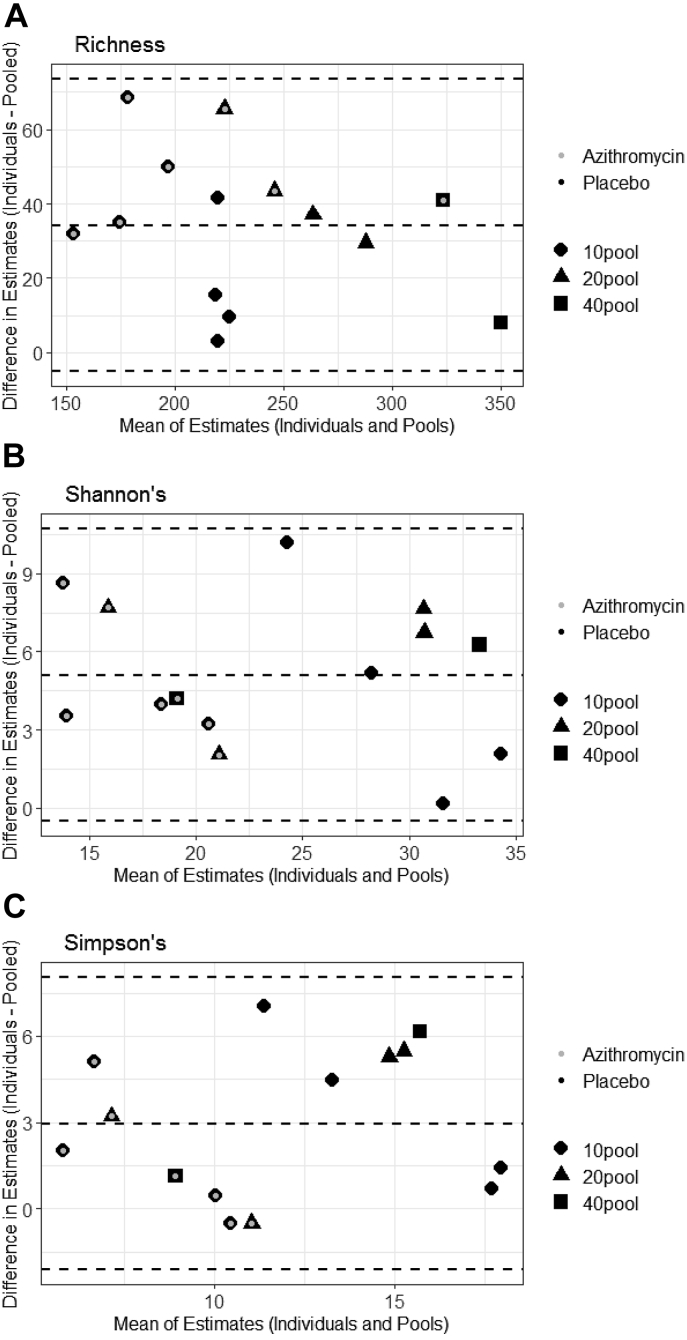
(A–C): Bland and Altman plot for gamma diversity estimates using individual samples and pooled samples. The middle dotted line shows the mean difference between the estimates (expected bias). The upper and lower dotted lines show 95% limits of agreement from −1.96SD to +1.96SD.

Comparisons of the two gamma diversity estimates across ranges of diversities are displayed in a Bland and Altman plot which illustrates the expected bias between the estimation methods (Fig. 2). The mean difference in the two estimation methods for richness was −34.25 genera (95% CI: −73.6 to 5.12), for Shannon's gamma diversity was −10.72 (95% CI: −5.12 to 0.50), and Simpson's diversity was −3.0 (95% CI: −8.05 to 2.13). All pooled samples and individual samples are within the 95% limits of agreement. In addition, we see no evidence of systematic differences or heteroscedasticity across the range of all three diversity indices.

**Fig. 3:**
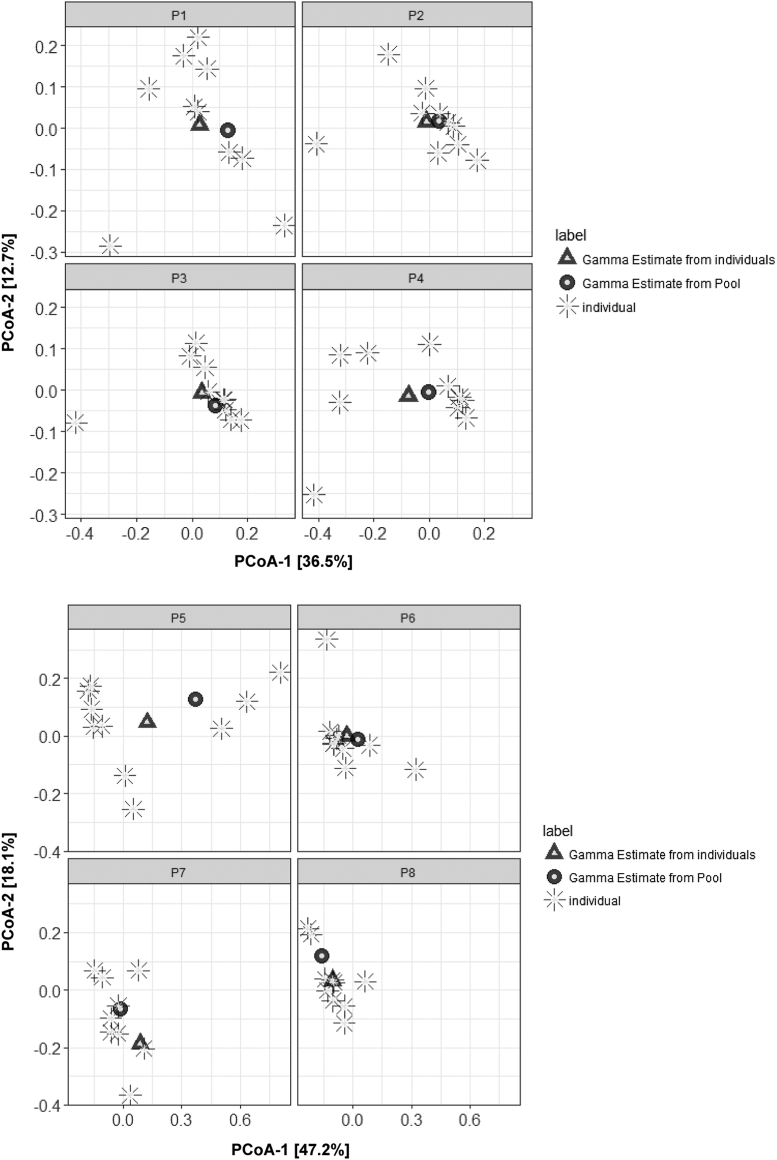
PCoA plots for community or gamma diversity estimates using individual samples (triangles), pooled samples (circles). Also plotted are alpha diversity of individual samples (stars) within the community. P1-P4 are communities treated with placebo and P5-P6 are treated with antibiotics. PCoA, principal coordinates analysis.

Principal coordinates analysis plots for community diversity estimates using individual samples (triangles), pooled samples (big circles), and individual samples (small circles) demonstrate the community-level microbiome as characterized by the different types of samples (Fig. 3). In principal coordinates analysis plots, points that are closer together represent microbial communities that are more similar in sequence composition. It is clear from Figure 3 that the gamma estimates are close and that they are estimating the alpha diversity of the individuals in the community.

## Discussion

Pooling with nucleic acid amplification testing has been used in a number of ways including targeted PCR to detect rare species, estimate overall community infection prevalence, single-nucleotide polymorphisms discovery, or estimate allele frequency [5,6,8]. Here, we estimate gamma diversity from pooled specimens for community-level association studies using microbiome diversity. We found this pooling method provides estimates of the community-level diversity that are highly correlated with gamma diversity estimates using individually sequenced samples. This method since has been used in practice to assess the effect of mass antibiotic treatment on community-level gamma diversity between treatment groups in a large CRT [18].

In any estimate, there will be bias. Here we consider the main sources of bias one should consider when pooling microbiome specimens to estimate gamma diversity, extracting equal amounts of DNA, PCR amplification of a pooled sample versus individual samples, and number of samples to pool. Before this experiment, we believed obtaining equal aliquots of DNA from component specimens might introduce too much bias to estimate gamma diversity as it’s been traditionally estimated. PCR amplification bias will also be introduced to the pooled sample estimation compared with the individual sample estimation differently. Arguably, pooling might be a better estimation of gamma diversity because PCR amplification bias is introduced once versus 10, 20, or 40 different times for individually processed samples. Finally, it should be noted that more individual specimens combined into a pooled sample reduces the estimation bias because as this number increases, alpha of a pool estimate approaches gamma of the individuals.

High-throughput sequencing approaches can be cost-prohibitive for large epidemiology trials, where hundreds if not thousands of samples are needed to be processed. As shown here, pooling can potentially cut the costs 10-fold for any given study. What is also hidden in these cost estimates is the human labor that is required for sample processing. While there are many steps in the library preparation for high-throughput sequencing that can be automated, the process remains time-consuming and requires supervision and human processing time where automation is not built in. It is expected that reagent and sequencing costs continue to decrease with time; the costs for human labor are likely to trend in the opposite direction. Therefore, the ability to pool samples without compromising statistical outcomes is particularly attractive from a financial standpoint.

Limitations of our study include that we only considered bacterial microorganisms, our sample size was small, and our population was an antibiotic naïve community with likely low heterogeneity among children's microbiomes compared with other communities. Pooling may not be a good strategy when microbiome sample heterogeneity is high, depending on your research question and the power of the study [19].

Gamma diversity is a relevant metric in CRTs. As previously shown, the microbiome changes daily and is dependent on the environment or one's surrounding community. Pooling microbiome samples before DNA amplification to estimate community level diversity is a viable and valuable measure to consider in population-level association research studies.
